# Familial Multiple Cavernous Malformation Syndrome: MR Features in This Uncommon but Silent Threat

**DOI:** 10.5334/jbr-btr.938

**Published:** 2016-03-21

**Authors:** Marc Mespreuve, Filip Vanhoenacker, Marc Lemmerling

**Affiliations:** 1 A.Z. St.-Maarten Mechelen, BE; 2 U.Z. Ghent, BE; 3 A.Z. St.-Lucas Ghent, BE

**Keywords:** Cerebral cavernous malformation, Familial cerebral cavernous malformation syndrome, MRI

## Abstract

Cerebral cavernous malformations (CCM) are vascular malformations in the brain and spinal cord. The familial form of cerebral cavernous malformation (FCCM) is uncommon. This autosomal dominant pathology mostly presents with seizures and focal neurological symptoms. Many persons affected by FCCM remain asymptomatic. However, acute hemorrhages may appear over time.

MRI demonstrates multiple focal regions of susceptibility induced signal loss, well seen on gradient-echo sequences (GRE) or even better on susceptibility-weighted imaging (SWI). The presence of a single CCM – especially in young persons – without history of FCCM does not exclude this diagnosis.

Some clinicians also advise an MRI of the spinal cord at the time of diagnosis to serve as a baseline and a control MRI of the brain every one to two years. MRI is certainly indicated in individuals with obvious new neurologic symptoms.

Symptomatic siblings should also undergo an MRI of the brain to determine presence, size, and location of the lesions. Even in asymptomatic siblings, a screening MRI may be considered, as there may be an increased risk of hemorrhage, spontaneous or due to the use of certain medications; the knowledge of the presence and the type of these lesions are important. Surgical removal of a CCM may be justified to prevent a life-threatening hemorrhage. Control MRI may reveal the postoperative outcome.

## Introduction

Cerebral cavernous malformations (CCM) are relatively common vascular malformations in the brain and spinal cord. The familial form of cerebral cavernous malformation (FCCM) is far less common, accounting for only a small minority of cavernous malformations but certainly underdiagnosed. Patients present with a variation of neurologic symptoms, and MR imaging is often the first diagnostic test. Our purpose is to review the MR features of FCCM.

## Definition

Cerebral cavernous malformations (CCM) or cerebral cavernous angiomas are vascular malformations in the brain and spinal cord. In sporadic cases, up to a third of such cavernous malformations are multiple.

Familial cerebral cavernous malformation syndrome (FCCM) is defined as the presence of multiple CCM (typically five or more) or the occurrence of CCM in at least two members of a family or the presence of a mutation in one of the three genes causing FCCM (Table 1) [1,2].

**Table 1:** Diagnostic criteria of familial cerebral cavernous malformation (FCCM) [1,2].

Diagnostic Criteria of FCCM (At Least 1 of the Following Criteria):

– the presence of multiple CCM (typically 5 or more)
– the occurrence of CCM in at least two members of a family
– the presence of a mutation in one of the three genes causing FCCM.

Due to the dynamic nature of CCM, new lesions may appear at a rate of between 0.2 and 0.4 lesions per patient-year [3]. This explains why older patients present with diffuse CCM, although young patients may present with already diffuse FCCM.

## Clinical Findings

The disease most commonly presents with seizures (38%–55%) and focal neurological deficits (35%–50%) [4]. A (recurrent) cerebral haemorrhage or a nonspecific headache are less frequently encountered symptoms. Exceptionally, a spontaneous paraplegia may occur. However 25–50 percent of individuals with CCM in general remain symptom free throughout their lives [5]. As many persons affected by FCCM remain asymptomatic, an underestimation of its frequency is very probable. Repeated minor bleeding from the lesions may cause degeneration. Resulting dementia and parkinsonism have been reported in patients with FCCM [6]. Autopsy revealed that even 90 percent of persons with a CCM never had symptoms during their lives [7].

Although cerebral cavernous malformations have been reported in children, the majority of patients present with symptoms between the second and fifth decades.

There is a coexistence of 33 percent between cavernous malformations and developmental venous anomalies [8,9]. Skin (9%), retinal (5%) and liver lesions have occasionally been reported. Only one of our patients had a cutaneous hemangioma [10].

## Histology

CCM are composed of closely clustered and enlarged capillary channels (called *caverns*) with a single layer of endothelium. The mature vessel wall elements are absent, as is the normal intervening brain parenchyma. They range from a few millimeters to several centimeters and can – due to their dynamic nature – increase (or sometimes decrease) in size and increase in number over time.

## Genetics

The suspicion of FCCM by physical examination, family history, and brain and spinal cord MRI may be confirmed by genetic testing. Three genes are known to cause mutations in FCCM: KRIT-1 (CCM-1), CCM-2, and PDCD-10 (CCM-3) [11]. At least one other unspecified gene (CCM-4) located on the long arm of chromosome three may also cause FCCM [12]. Moreover, an association of parenchymal FCCM and dural-based meningiomas has also been reported in PDCD10-mutated individuals [13].

FCCM is an autosomal dominant disease. Each child of an individual with FCCM has a 50 percent chance of inheriting the mutation. A much higher incidence of FCCM has been reported in Hispanic-American individuals of Mexican descent [3,14], in which the proportion of familial cases (in the general population only about 20%) is estimated to be up to 50 percent. These persons seem to be related to a common ancestor with a mutation in the KRIT-1 gene [14].

## MR Features

**Figure 1:**
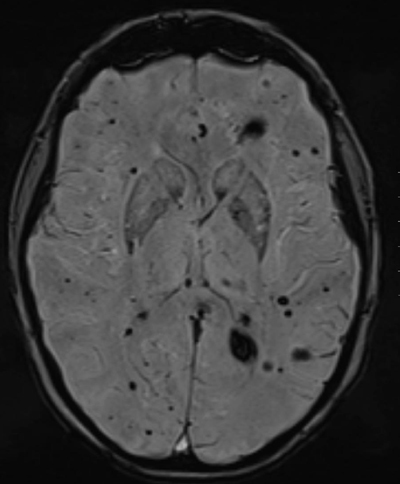
FCCM. Axial SWI. Bilateral diffuse presence of multiple focal regions of susceptibility-induced signal loss of variable size. Blooming is most obvious around the two larger lesions.

In typical cases, MRI shows multiple bilateral and diffuse focal regions of susceptibility induced signal loss (Figure 1) of variable size, well seen on gradient-echo (GRE) sequences, or even better on susceptibility-weighted imaging (SWI). GRE reveal up to triple the number of lesions seen on SE T2-WI, and SWI even reveals an additional tripling [15,16]. They range in size from a few millimeters to several centimeters in diameter. However, all T2* sensitive sequences result in a degree of blooming, seen as a hazy halo of signal loss around the lesion, and in an overestimation of the actual size of the lesions. Blooming is most obvious around larger lesions (Figure 1).

**Figure 2:**
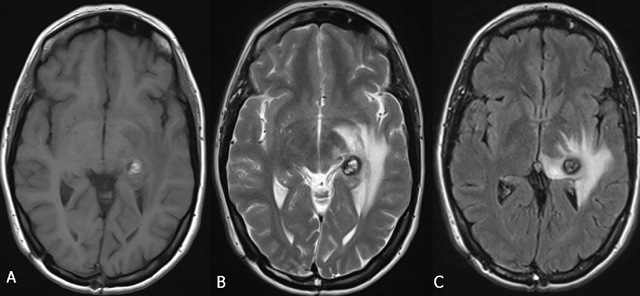
FCCM Type 1 lesion. Axial SE T1-WI (A), TSE T2-WI (B), and FLAIR (C). Hyperintense core on SE T1-WI (A) and SE T2-WI (B) suggests recent hemorrhage. Peripheral oedema is best seen on FLAIR image (C).

**Figure 3:**
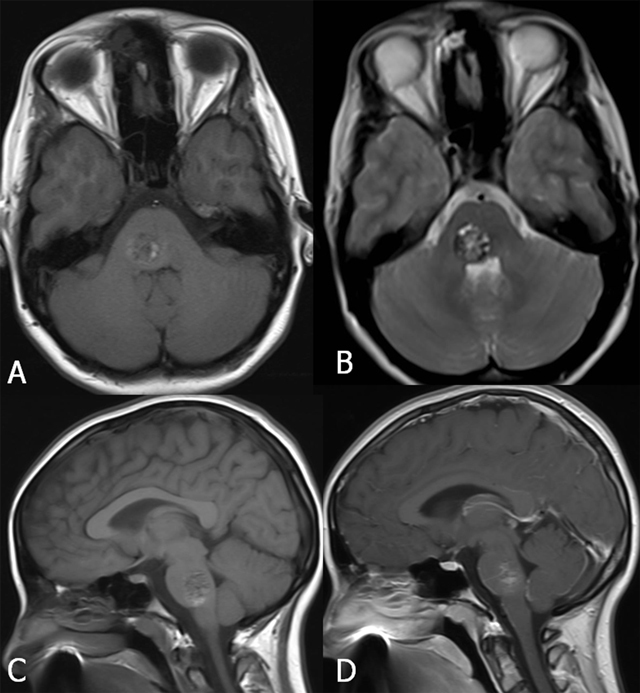
FCCM Type 2 lesion located in the brainstem. Axial SE T1-WI (A) and TSE T2-WI (B), sagittal SE T1-WI before (C) and after gadolinium contrast (D). Twenty-year-old patient with known FCCM (daughter of a Krit-I positive patient with FCCM), who presented with only two CCM, of whom one large lesion was located in the brainstem. The lesion shows a reticulated mixed signal core on SE T1-WI and reticulated mixed signal core with surrounding hypointense rim on SE T2-WI.

**Figure 4:**
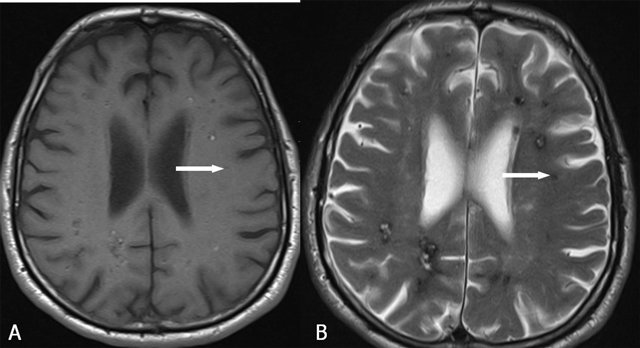
FCCM Type 3 lesion (arrows). Axial SE T1-WI (A) and TSE T2-WI (B). Hypointense lesion on SE T1-WI (A) and hypointense lesion with surrounding hypointense rim on TSE T2-WI (B).

**Figure 5:**
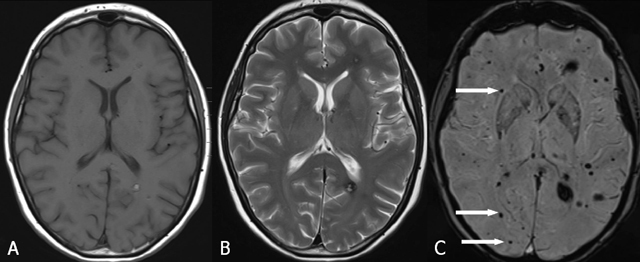
FCCM Type 4 lesion. Axial SE T1-WI (A), TSE T2-WI (B) and SWI (C). Multiple punctuate hypointense lesions (arrows) on SWI (C), not seen on SE T1-WI (A), nor on TSE T2-WI (B).

On MRI, four types of lesions are found (Table 2) [15]. A type 1 lesion with hyperintense core on SE T1-WI and SE T2-WI suggests recent hemorrhage (Figure 2A and 2B). Peripheral oedema is best seen on FLAIR-weighted images (Figure 2C). A reticulated mixed signal core on SE T1-WI and reticulated mixed signal core with surrounding hypointense rim on SE T2-WI is compatible with a type 2 lesion (Figure 3). Hypointense lesion on SE T1-WI and hypointense lesion with hypointense rim SE T2-WI and blooming on GRE-T2* are referred to as type 3 lesions (Figure 4), whereas punctuate hypointense lesions on GRE-T2* or SWI, not seen on SE T1-WI, nor on SE T2-WI, are designated as type 4 lesions (Figure 5).

**Table 2:** FCCM types of lesions on different MRI sequences and correlation with clinical correlation and histopathology [11].

FCCM	SE T1-WI	(T)SE T2-WI	GRE-T2*	SWI	Clinical Correlation	Histopathology

Type 1	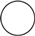	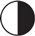			– Acute hemorrhage – High frequency of bleeding relapse	Subacute hemorrhage
Type 2	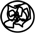	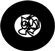				Lesions with hemorrhages and thromboses of varying ages
Type 3	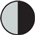	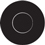	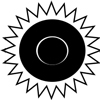	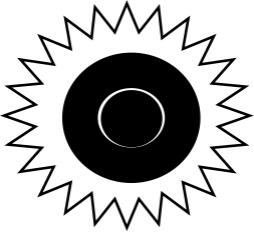	possibly represent new lesions	Chronic hemorrhage with hemosiderin within and around the lesion
Type 4	Not visible	Not visible	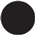	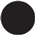		Tiny CCM or telangiectasia
**LEGEND**						
		
Density in relation to the surrounding brain parenchyma	Part and form of the lesion				
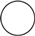 Hyperintense	Punctate lesion	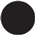		
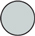 Isointense	Core	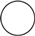		
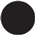 Hypointense	Rim	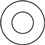		
	Surroundings (blooming)	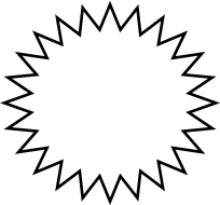		

*Note*: SE = spin echo, GRE = gradient echo, SWI = susceptibility-weighted imaging.

The identification of type 1 lesions, with a high frequency of recurrent bleeding, and type 4 lesions, which possibly represent new lesions, is important. The lesions may demonstrate changing signal. High signal on SE T1- and T2-WI centrally with a variable degree of perilesional oedema, best seen on fluid attenuated inversion recovery (FLAIR) sequences, suggests a more recent bleeding. The clinical significance of small lesions seen on MRI remains unknown. However, detection of multiple lesions is helpful in distinguishing CCM from FCCM. Moreover, the meticulous evaluation of the number, location, and size of the lesions is important, as there may be an increased risk of hemorrhage with certain analgesic medications (nonsteroidal anti-inflammatory drugs) and the frequently used acetylsalicylic acid. The risk-benefit of medications that increase the frequency of hemorrhage (heparin and coumarin-type drugs) should be weighed thoroughly before use.

**Figure 6:**
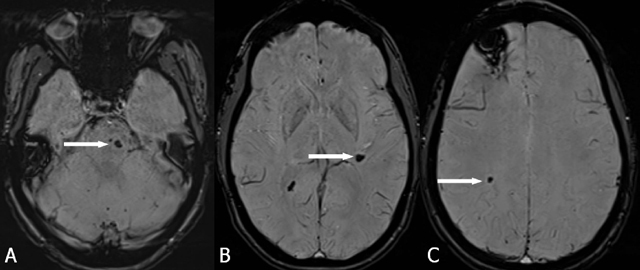
These axial SWI respectively at the level of the brainstem (A), basal ganglia (B), and semioval center (C), performed on the occasion of a familial screening in a 29-year-old daughter of a patient (figure 1, Krit-I positive) with FCCM shows already multiple (only the largest lesion at the different levels marked with an arrow) infratentorial and supratentorial cavernomas with typical signal loss.

CCM can be multiple (in up to a third of the patients). However, in FCCM the number of cavernomas is higher, typically five or more [3]. Hundreds of lesions may be identified, normally increasing with the person’s age. However, young patients may already show numerous CCM (Figure 6).

**Figure 7:**
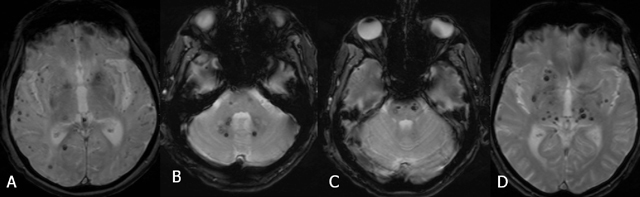
FCCM location in the brain in two patients ((A) and (B–D)). Axial SWI. Presence of multiple (A) supratentorial and (B–C) infra- and (D) supratentorial lesions.

**Figure 8:**
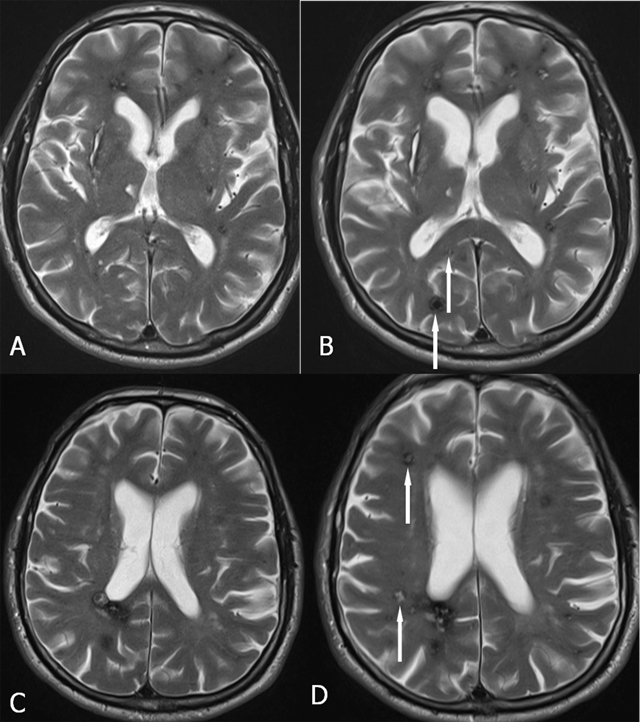
Evolution of the number of lesions in FCCM. Axial TSE T2-WI (A and C in 2009; B and D in 2013). Appearance of at least four new lesions over time (arrows).

Lesions may be located either supratentorially (75%) or infratentorially (25%) [3] (Figure 7). Almost half of the infratentorial lesions occur in the brainstem, and these are frequently associated with symptoms related to hemorrhage. Cavernous malformation can lead to death from severe intracranial hemorrhage, particularly when located in the brainstem [17]. MRI is a useful tool to assess evolution of the number of CCM over time (Figure 8). Screening MRI brain examinations of family members is useful to detect infra- and supratentorial lesions in the brain (Figure 6).

Lesions may be detected only occasionally in the spinal cord (about 5%). Secondary superficial siderosis [18,19] is exceptional in superficially located lesions. The presence of a single (or less than five) CCM – especially in young persons – without a history of FCCM does not exclude this diagnosis at all (Figure 3). MRI evaluation over time shows the appearance of new lesions and of acute (often asymptomatic) hemorrhages (0.7% lesions per patient-year) that change in signal intensity and increase in size over time [20]. In children, hemorrhage with an aggressive presentation may be more likely.

Intravenous gadolinium contrast administration is not required for the diagnosis of (F)CCM, but contrast-enhanced MRI may be useful in identifying more complex vascular malformations which are sometimes associated with CCM. It may also help to distinguish CCM from other types of vascular brain malformations such as capillary telangiectasia, aneurysms, and arteriovenous malformations and are indicated if surgical resection is considered (detection of the venous drainage in order to preserve it). CCM are rarely visualized on angiography because of the small size of the afferent vessels, the presence of thrombosis, and the relatively low flow in a CCM.

Some clinicians advocate MRI of the spinal cord at the time of diagnosis to serve as a baseline for future follow-up. A control MRI of the brain every one to two years (GRE or SWI) is indicated, also in individuals with obvious new neurologic symptoms. Interpretation may be difficult, as new lesions may be asymptomatic. (Symptomatic) siblings should undergo MRI of the brain to determine presence, size, and location of lesions.

Surgical removal of lesions associated with seizures or focal deficits from recurrent hemorrhage or mass effect may be justified, even when a large number of other lesions is present. Follow-up MRI may evaluate postoperative outcome (lesion resection, bleeding, loss of brain parenchyma).

## Differential Diagnosis

All other causes of cerebral microhaemorrhages are to be considered.

**Figure 9:**
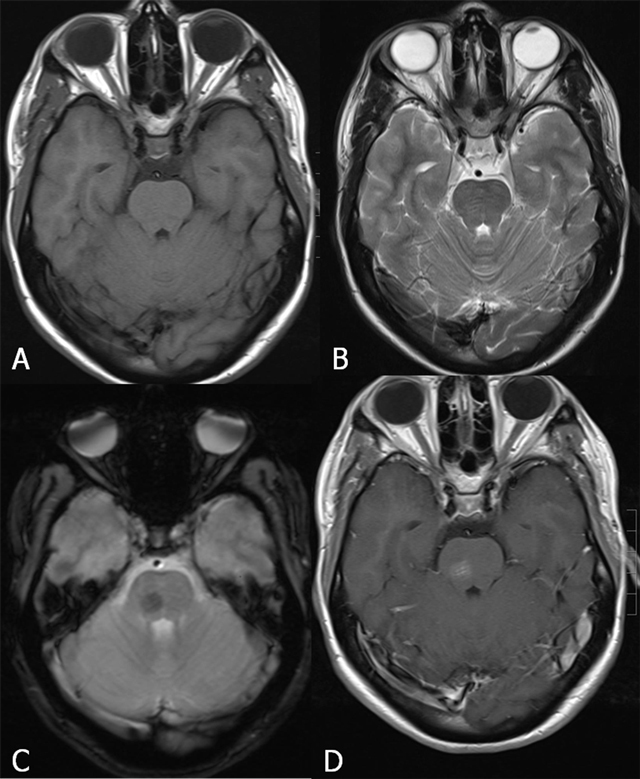
Capillary telangiectasia in the brainstem (arrows) Axial SE T1-WI (A), TSE T2-WI (B), GRE-T2*(C) and SE T1-WI after gadolinium contrast (D). The lesion is not visualized on T1-WI (A). On T2-WI, there is a slightly increased signal intensity (B), whereas the lesion is of low signal on GRE-T2*(C). After gadolinium contrast (D), there is a faint enhancement in a brush-like pattern.

**Figure 10:**
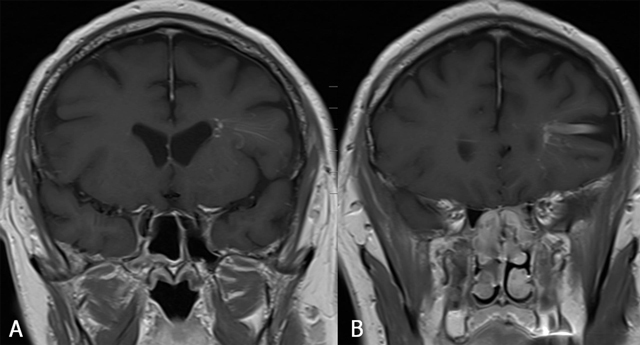
Arteriovenous malformation. Coronal SE T1-WI. Typical arterial (A) and venous component.

Other types of vascular brain malformations consist of capillary telangiectasias (Figure 9), aneurysms, and arteriovenous malformations (Figure 10).

**Figure 11:**
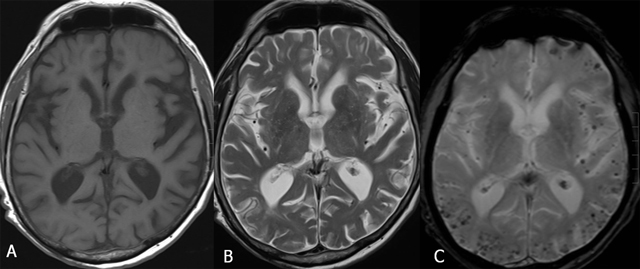
Cerebral amyloid angiopathy. Axial SE T1-WI (A), TSE T2-WI (B) and GRE-T2*(C). Numerous small foci of uniform size and susceptibility-induced signal loss with a predilection for the peripheral subcortical white matter, best seen on GRE-T2* (or SWI).

**Figure 12:**
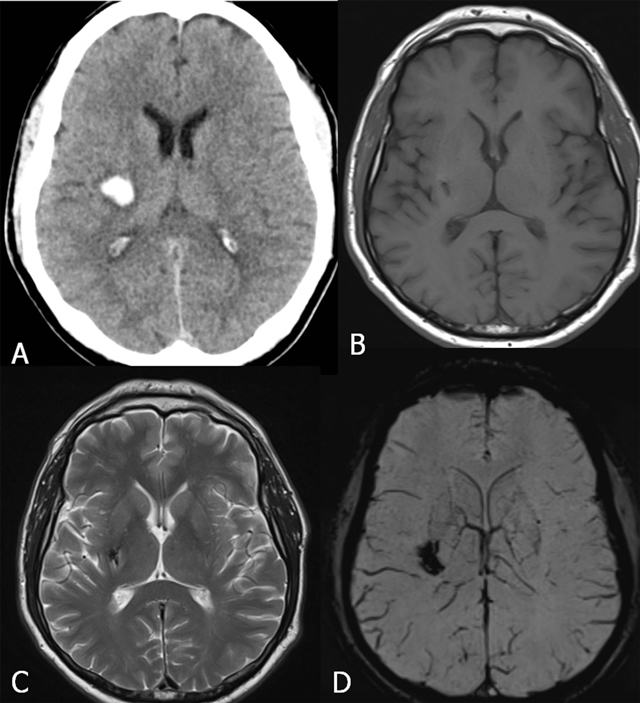
Hypertensive central hemorrhage in patient with severe hypertension. CT (acute fase) (A) and follow-up MRI with SE T1-WI (B), TSE T2-WI (c) and SWI (D). Acute cerebral hemorrhage (A). Parenchymal defect (B) and focus of low signal with susceptibility-induced signal loss (C, D) best seen on SWI.

In cerebral amyloid angiopathy (Figure 11), numerous small foci may have a similar imaging appearance, with a predilection, however, for the peripheral subcortical white matter. There may also be a history of prior (larger) lobar haemorrhage or superficial siderosis.

(Chronic) hypertensive encephalopathy has a predilection for the region of the basal ganglia (Figure 12).

**Figure 13:**
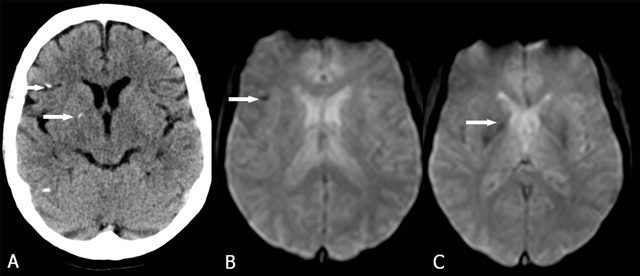
Diffuse axional injury. CT (A) and GRE-T2*(B, C). Multiple small foci of high density (A) corresponding with (arrows) small foci of low signal intensity (B, C) at the borders between the cortical grey and white matter.

In diffuse axonal injury (Figure 13), there is a history of severe trauma, and the lesions have a typical distribution at the borders between the cortical grey matter and the underlying white matter.

Neurocysticercosis lesions are more uniform and smaller in size; they may calcify by time and are typically more regularly delineated. CT or quantitative susceptibility mapping [21] on MRI may confirm the calcifications.

Radiation-induced cavernous malformations are radiologically and even pathologically indistinguishable from FCCM [22]. Anamnesis of previous radiation (frequently during childhood) may reveal the diagnosis.

**Figure 14:**
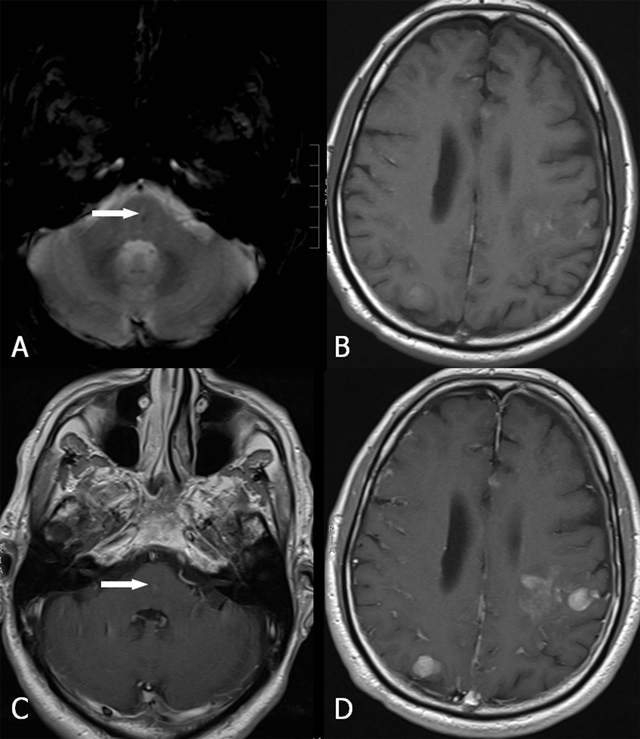
Hemorrhagic metastases. GRE-T2*(A), SE T1-WI (B), and SE T1-WI (C, D) after contrast. Small focus of low signal (A) in the pons (arrow) with faint contrast enhancement (C) (arrow). Presence of multiple, supratentorial, nodular hemorrhagic (B), and enhancing lesions (D).

More rare are hemorrhagic metastases (Figure 14) and de novo bleeding in cerebral vasculitis and radiation vasculopathy.

## Therapy

**Figure 15:**
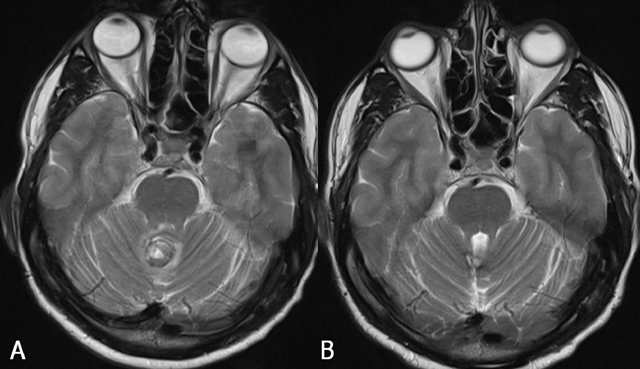
Resection of a cavernoma: pre- and postoperative MRI. Axial TSE T2-WI. A large cavernoma (A) is present in the vermis of the cerebellum. After resection (B), there is only a minimal loss of brain parenchyma and a small marginal region with susceptibility artifacts.

Microsurgical removal of a CCM lesion may be justified if associated with seizures or focal deficits from recurrent hemorrhage or mass effect. To reveal the structural and the functional abnormalities of a seizure, the data from EEG, MRI, and SPECT may be focused and integrated. The spatial relationships may be demonstrated by co-registering images of the abnormalities on the MRI. A recent technique of subtraction known as ictal SPECT co-registered to MRI (SISCOM) may reveal a hyperperfusion focus. The SISCOM focus and its relationship to the brain may serve as a map for subsequent surgical resection. A follow-up MRI is an excellent technique to monitor the postoperative outcome. MRI control after surgery in a patient with CCM traditionally shows complete resection, a minimal loss of brain parenchyma, and a small marginal region of susceptibility artifact (Figure 15).

## Conclusion

The familial form of cerebral cavernous malformation is uncommon. However, as this autosomal dominant pathology presents with a variety of neurological symptoms and as, on the other hand, many persons affected by FCCM remain asymptomatic, radiologists should include FCCM in their differential diagnosis. The presence of a single CCM in an individual, even without a history of FCCM, does not exclude this diagnosis, and a control MRI of the brain after one to two years is advised. MRI with gradient-echo sequences (GRE) or even preferably susceptibility-weighted imaging (SWI) of the brain is indicated for the diagnosis and to serve as a baseline examination. The identification of the type of lesions is important to evaluate the risk of bleeding relapse. The knowledge of the presence of FCCM is important, as there may be an increased risk of hemorrhage with certain medications. Control MRI of the brain is indicated over time, certainly with obvious new neurologic symptoms, and after surgical removal. Symptomatic and possibly even asymptomatic siblings may also undergo MRI of the brain to determine presence, size, and location of the lesions.

## Competing Interests

The authors declare that they have no competing interests.
